# Examining the Effects of Stiffness and Mass Difference on the Thermal Interface Conductance Between Lennard-Jones Solids

**DOI:** 10.1038/srep18361

**Published:** 2015-12-17

**Authors:** Kiarash Gordiz, Asegun Henry

**Affiliations:** 1 George W. Woodruff School of Mechanical Engineering, Georgia Institute of Technology, Atlanta GA, 30332; 2 School of Materials Science and Engineering, Georgia Institute of Technology, Atlanta GA, 30332.

## Abstract

To date, the established methods that describe thermal interface conductance (TIC) and include mode-level dependence have not included anharmonicity. The current intuition is therefore based on the behavior in the harmonic limit, whereby the extent of overlap in the bulk phonon density of states (DoS) (e.g., frequency overlap) dictates the TIC and more frequency overlap leads to higher TIC. Here, we study over 2,000 interfaces described by the Lennard-Jones potential using equilibrium molecular dynamics simulations, whereby we systematically change the mass and stiffness of each side. We show that the trends in TIC do not generally follow that of the bulk phonon DoS overlap, but instead more closely follow the vibrational power spectrum overlap for the interfacial atoms. We then identify the frequency overlap in the interfacial power spectra as an improved descriptor for understanding the qualitative trends in TIC. Although improved, the results show that the basic intuition of frequency overlap is still insufficient to explain all of the features, as the remaining variations are shown to arise from anharmonicity, which is a critical effect to include in interface calculations above cryogenic temperatures.

Thermal transport properties in nanostructures are often significantly impacted by the introduction of interfaces to the system^1^,^2^,^3^. Unlike the study of thermal conductivity in crystalline materials that has advanced tremendously in recent years^4^, many questions still remain regarding heat conduction through an interface between dissimilar materials. It is now possible to not only calculate thermal conductivity for existing crystalline materials from different methods^5^ including first-principles^4^,^6^, but one can even predict the thermal conductivity of pure crystalline materials that have yet to be synthesized and experimentally measured^7^. For the study of heat transfer across interfaces, however, the situation is different, as no excellent agreement with experiment over a wide range of temperatures or for various material systems has ever been reported^8^,^9^,^10^,^11^,^12^,^13^,^14^,^15^.

The picture that is ubiquitously used for thermal interface conductance (TIC) is based on phonon gas model (PGM)^16^, which casts the problem as a transmission problem for phonons impinging on the interface: 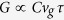. Here, 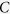 and 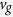 are the heat capacity and group velocity of a phonon that impinges on an interface. The phonon energy can then either be transmitted or reflected with a specific transmission probability defined as 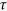. The PGM treatment of TIC lumps all of the physics associated with the nature of the other material, the quality of the interface and the nature of the chemical bonding/interactions (e.g., everything other than heat capacity and group velocity) into the transmission probability 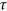. Thus, different physical effects such as interface roughness, inter-diffusion, stress, and dislocations all manifest through 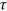. It is this single descriptor that encompasses all of the dynamics of the interface and it is the only place where the properties of the other material (e.g., its stiffness, chemical composition, atomic level topography) enter the calculations.

To date, the methods that have been developed to describe 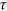, or the mode level TIC contributions have been unable to include anharmonicity, with the exception of Hopkins and coworkers for the Diffuse Mismatch Model (DMM)^11^ and Mingo’s work developing the Atomistic Green’s Function (AGF) method^15^. Nonetheless, the only guiding intuition we have is still based on the harmonic limit, where only elastic phonon interactions can occur, which restricts a mode with frequency 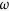 to interactions with other modes of the same frequency. It is then intuitive to reason that if more modes of a given frequency are available on the other side, the probability of phonon transmission is higher and this general trend is reproduced by all of the previous methods that elucidate the mode level contributions^8^,^17^,^18^. This basic principle gives rise to the idea that high TIC occurs when a large degree of frequency overlap between the phonon density of states (DoS) exists between the two materials. However, this intuition is only rigorously correct as one approaches absolute zero. At higher temperatures, several new and emerging methods have shown that anharmonic effects (e.g., inelastic interactions) become non-negligible at temperatures as low as 10 K^19^,^20^. Nonetheless, even though anharmonicity is important, a remaining issue is the fact that the guiding intuition of whether or not frequency overlap is a truly useful descriptor for TIC, has yet to be verified independently i.e., by using an independent method to evaluate TIC that is not based on the same basic framework, such as an experiment or another type of calculation. Here, we used equilibrium molecular dynamics (EMD) to calculate TIC independent of models that yield results consistent with frequency overlap as a means of evaluating the conventional intuition. For simplicity, we used the Lennard-Jones (LJ) potential to systematically change the mass mismatch and stiffness mismatch at the interface, which then changes the degree of frequency overlap. The degree of overlap is then evaluated by calculating the phonon DoS for the atoms in each respective system and the TIC is calculated using DMM to assess if there is qualitative agreement with the EMD results.

## Model Details

The systems are constructed from two face-centered-cubic (FCC) LJ solids that are lattice matched, which is a structure that has been used extensively in other interfacial heat transfer studies^19^,^21^,^22^,^23^. The LJ interatomic potential is taken as,


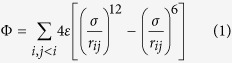


where 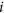 and 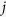 are atom indices and 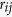 is the distance between atoms 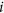 and 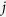. We have three tunable parameters on each side of the interface: the depth of the potential well (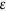) (e.g., the stiffness), the location of the potential well minimum 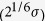, and the mass (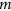) of the atoms. For any set of LJ parameters for the materials 1 and 2 forming the interface (e.g., 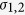 and 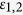), we used mixing rules to define the LJ parameters for the cross-species interactions: 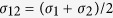, 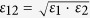
24. As a result, the quantitative values for TIC and qualitative trends for TIC obtained herein are specific to these choices. However, as will be shown later, the conclusions are interesting and instructive for our intuition, and it is straightforward to reason that the conclusions are likely to still have meaning and relevance for more technologically relevant interfaces. Hence, the interest here in such a simplified system is to enable a systematic and independent evaluation of the effects of mass and stiffness mismatch. For other, more realistic interatomic potential choices, the interactions are typically more complicated and most often the stiffness and lattice parameter cannot be changed independently. This can make it difficult to isolate the effect of stiffness itself (e.g., over a large range of stiffness values), without inducing other effects such as lattice mismatch and its associated stress and defects. Here, however, by using LJ solids, we can isolate the effect of stiffness, by keeping the two sides lattice-matched. This is accomplished by simply selecting equal values of 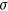 for the two sides of the interface^22^, which then allows us to independently explore the effects of 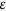 and 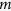 on the TIC.

**Figure 1:**
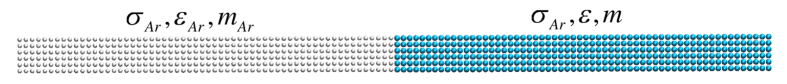
Schematic of our model structure: the interface of two LJ solids. Different interfaces will be formed by keeping the left side of the interface constant and equal to solid argon (indicated by subscript Ar) and modifying 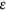 and 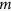 on the right side of the interface.

A schematic of the model structures is shown in Fig. 1. We start from the case of having solid argon on both sides of the interface. LJ parameters and mass of argon atom (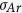, 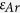, and 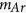) are chosen from Ref. 24. Then, keeping all the parameters constant on one side of the interface (e.g., solid Ar), we independently increase 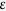 and 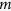 on the other side of the interface from 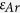 and 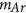 to 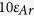 and 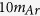. In this way, we examine the impact of a 10X difference in stiffness and mass. This allows us to generate many interfaces all having solid argon on one side and another solid with different properties on the other. Henceforth, we will refer to these two sides as the *“constant”* side and the *“varying”* side respectively. This allows us to study a broad range of distinct interfaces instead of evaluating interfaces by modifying only one parameter as has been done previously^19^,^21^,^22^,^23^. The results can then be examined as two-dimensional maps of 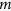 and 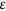 (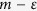 map) with x- and y-axis representing the changes in 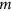 and 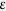 on the *varying* side and the height (z-axis) equal to the property of interest (e.g., TIC or frequency overlap). Each point on the 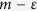 map therefore represents a distinct structure and interface. In this study, we changed the values of 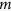 and 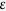 along the 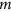 and 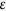 axes with a resolution of 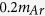 and 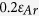, respectively, which resulted in a total of 2116 distinct interfaces. We considered 3 × 3 × 20 (x, y, z) FCC unit cell systems, for both the *constant* and *varying* sides. Larger cross sections up to 5 × 5 unit cells and longer systems up to 40 unit cells exhibited less than 5% difference from the 3 × 3 × 20 structures suggesting the 3 × 3 × 20 structures are representative of the limiting behavior of contact between two bulk crystals. The z-direction is chosen along the [100] crystallographic direction and is perpendicular to the planar interface. Periodic boundary conditions are applied in all three dimensions.

The LJ cutoff radius is chosen to be equal to 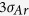 and a small time step of 0.15 fs is used for all EMD simulations to properly model the dynamics of the highest frequency systems on the 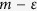 map. To reach equilibrium, we simulated each structure successively in the isothermal-isobaric (NPT) ensemble for 0.5 ns and in canonical (NVT) ensemble for another 0.5 ns. Then, using Eq. 3, we outputted the values of interfacial heat flow under the microcanonical (NVE) ensemble for 3 ns and used Eq. 2 to calculate the TIC value. We averaged the results over 20 ensembles^25^ with different initial random velocities, which reduced the statistical variation to less than 4% for all the interfaces on the 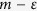 map (a total of 42,320 independent EMD simulations). All simulations were conducted using the Large-scale Atomic/Molecular Massively Parallel Simulator (LAMMPS) package^26^ and the force routine was modified to include the interfacial heat flux calculation concurrently with the trajectory evolution.

In EMD, the TIC is calculated from the autocorrelation function of the interfacial heat flow, using the fluctuation-dissipation theorem^27^ and can be written as^28^,^29^,


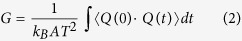


where 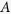, 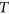, 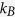, and 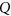 are the cross-section, simulation temperature, Boltzmann constant, and instantaneous interfacial heat flow across the interface, respectively, and 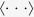 represents the ensemble average. For a pair-wise inter-atomic potential such as LJ, 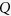 can be defined as^28^,


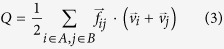


where 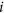 and 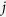 represent atomic indices, 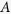 and 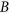 represent the two sides of the interface, 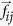 is the force from atom 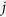 on atom 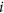, and 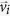 and 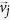 are the velocities of the atoms 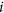 and 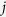.

## Results and Discussion

**Figure 2:**
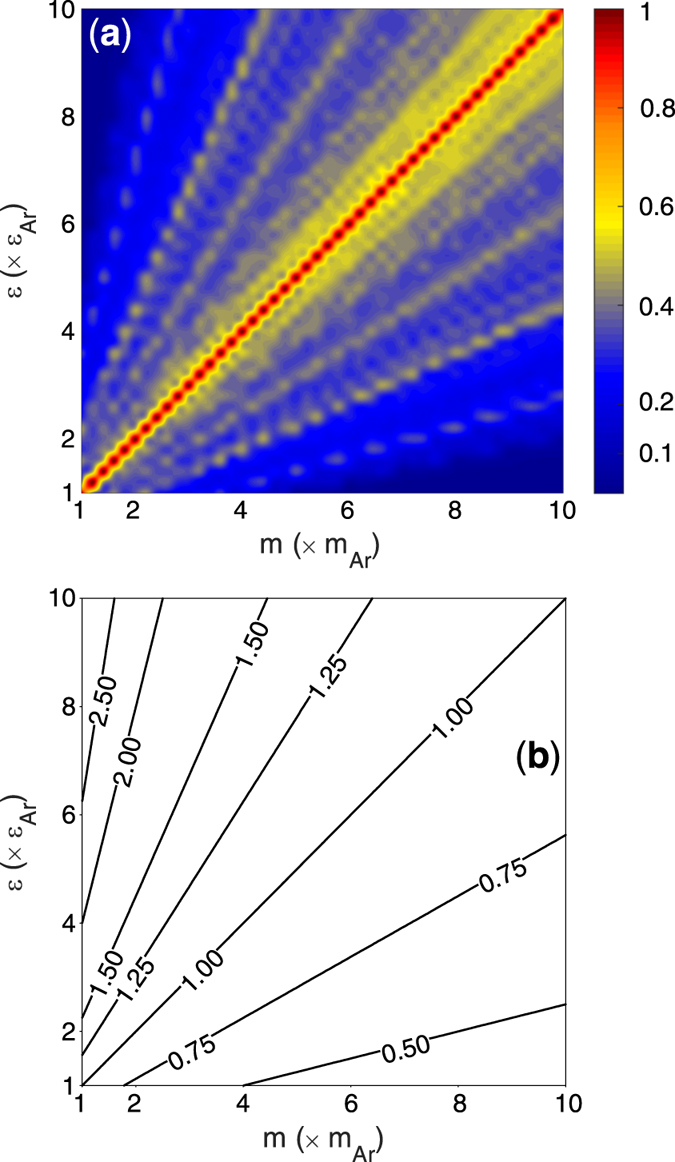
(**a**) Degree of overlap between the DoS of the two sides of the interface and (**b**) ratio of the characteristic frequency of the *varying* side to the one of the *constant* side.

We first determined the frequency content of each structure by lattice dynamics (LD) calculations^30^. No negative frequencies were observed for any of the structures indicating that all of them are stable^30^. Using the LD derived DoS for the bulk of each material (*constant* and *varying*) we quantified the degree of overlap between the DoS of the two sides as^31^,


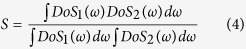


where 1 and 2 subscripts represent *constant* and *varying* sides, respectively. The degree of DoS overlap for the entire 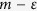 interval under consideration is shown in Fig. 2a. It can be seen that the maximum values of DoS overlap between the two sides are present along the diagonal, which is expected since individual oscillator frequencies scale as 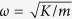, where for a monatomic LJ solid 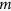 is the mass of the atoms in the system and 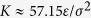 is the approximate characteristic spring constant between two first nearest neighbor atoms in the FCC lattice. The systems corresponding to the diagonal in Fig. 2a have the same 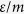 ratio as the *constant* side therefore these systems have the same characteristic frequencies and identical dispersion curves, which leads to the maximum DoS overlap. The ratio of the characteristic frequency of the *varying* side to the characteristic frequency of the *constant* side is also shown in Fig. 2b and is another way of visualizing the trends in DoS overlap (Fig. 2a). Figure 2a,b suggest that the highest contrast in vibrational properties between the bulk of the two sides occurs when the stiffness is the same with maximum mass difference (bottom right) and when the mass is the same and but the stiffness difference is maximum (top left). The guiding intuition of frequency overlap would then suggest that these systems should exhibit the lowest values of TIC for the interval considered.

**Figure 3:**
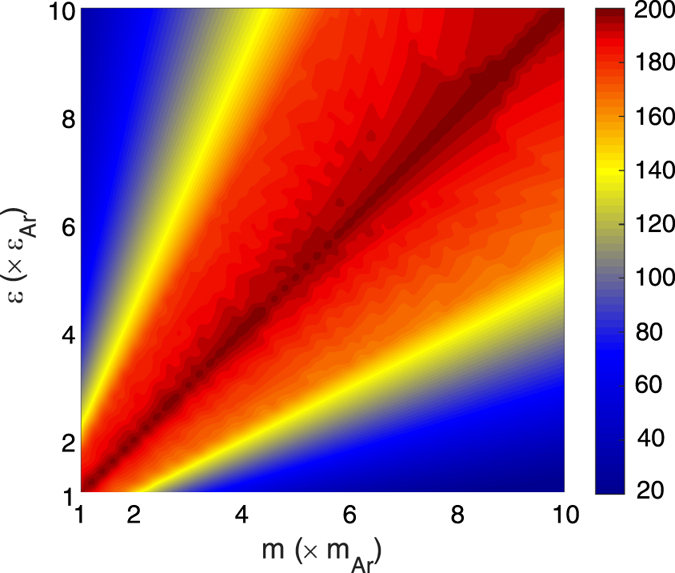
Values of TIC calculated at T = 40 K using DMM. TIC values have units of 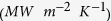.

We then used the DMM to calculate the values of TIC for the interfaces on the 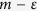 map (see Fig. 3). The details associated with using the DMM are available in the original work of Swartz and Pohl^9^ and works by Hopkins *et al.*^10^,^11^,^32^. Here, we used the DMM with the following assumptions: (1) isotropic properties for both sides of the interface, (2) Debye model for the phonon dispersion and DoS, and (3) elastic interactions between phonons across the interface. In addition, the TIC integral in the DMM should be performed on the softer side of the interface for all the structures on the 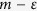 map and we evaluated the TIC values at T = 40 K. It should be mentioned that solid argon has a melting temperature of around 86 K and it is the softest structure among all of the solids considered. Therefore, none of the structures melt at T = 40 K. It should be noted that, even the approaches that can incorporate anharmonic interactions into DMM calculations^11^ would not yield qualitatively different predictions, since all of them are based on the vibrational modes calculated from the bulk of the materials. Even if we relax all of our earlier assumptions associated with performing the DMM calculations, the TIC for systems along the diagonal will not change. TIC remains constant because DMM fundamentally utilizes the vibrational information of the bulk of the materials, which are indeed the same for both sides in systems located on the diagonal. Therefore, the DMM in any of its current incarnations does not yield different predictions for drastically different materials that ultimately have the same phonon dispersion.

**Figure 4:**
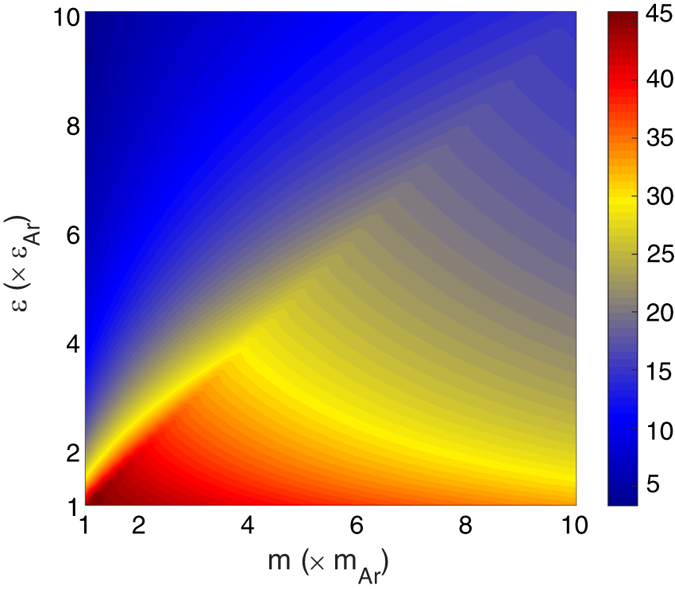
Values of TIC calculated at T = 40 K using AMM. TIC values have units of (MW m^–2^ K^–1^).

It should be noted that one should also consider the Acoustic Mismatch Model (AMM) since the interfaces are smooth. Fundamentally, the AMM is arguably more applicable than the DMM, because for such smooth interfaces one also has to take into account momentum conservation in the directions parallel to the interface^33^. Thus we also examine the AMM, as we have calculated the TIC values it predicts on the 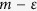 map in Fig. 4. The details associated with usage of the AMM can be found in Ref. 34. In Fig. 4 the AMM trend is drastically different from the DMM calculations (Fig. 3), which signifies the fundamental contrast between the two approaches. Interestingly, although the AMM technique is still based on the bulk vibrational information^34^, unlike the DMM approach, it can distinguish the different interfaces located on the diagonal of the 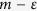 map. The reason is that the definition of transmission coefficient in AMM is based on the acoustic impedance (i.e., 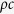, where 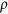 is the density and 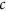 is the speed of sound in the material), and because of the large difference in mass on the 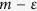 map, there is a large difference in density. As a result the AMM predicts that the acoustic impedance and therefore the TIC should vary along the diagonal of the 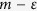 map.

**Figure 5:**
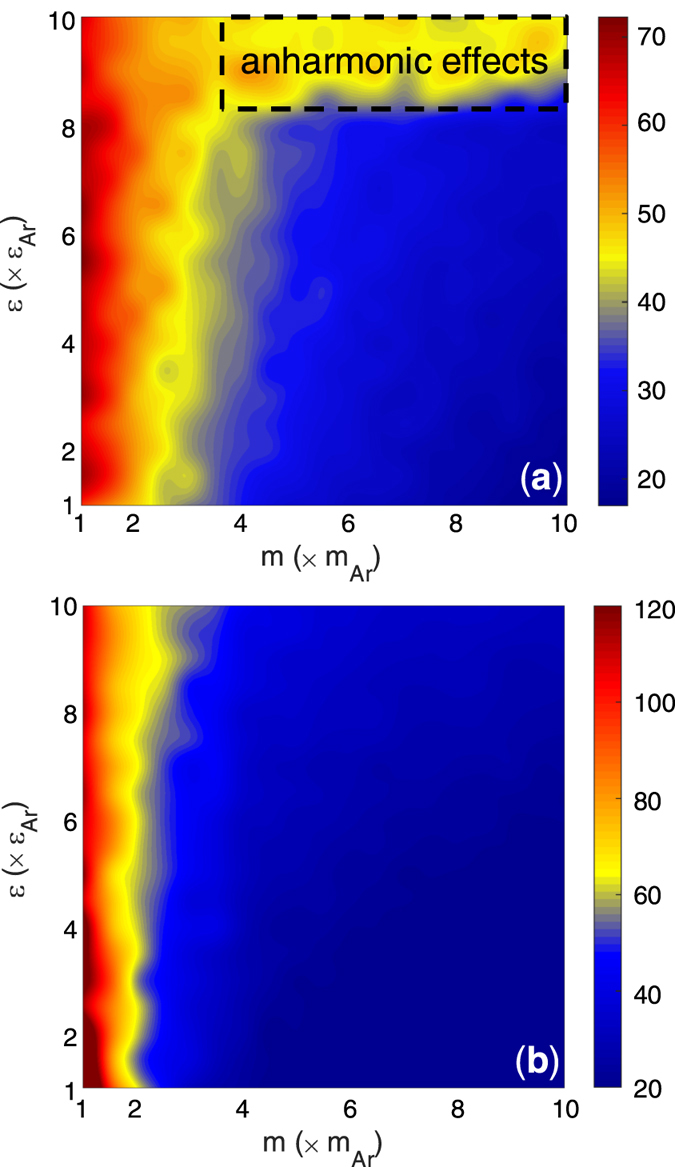
Values of TIC calculated using the EMD simulations at (**a**) T = 40 K, and (**b**) T = 5 K. TIC values have units of (MW m^–2^ K^–1^). The region that exhibits the strongest anharmonic effects is illustrated in panel (**a**) through the dashed rectangular region.

The EMD values of TIC are shown in Fig. 5a and they are in qualitative disagreement with the guiding intuition obtained from both frequency overlap analysis (Figs 2 and 3) and the AMM calculations (Fig. 4). This disagreement raises significant questions around the applicability of the harmonic analyses based on the bulk vibrational characteristics of materials. Clearly, utilizing the bulk vibrational information from sides of the interface in DMM and AMM fails to properly describe the trends in TIC for the systems on the 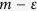 map. One interesting insight that can in general be obtained from the observed trends in Fig. 5a is that the mass-mismatch between the two sides of the interface seems to have a much stronger effect on the TIC than the stiffness mismatch. The significance of mass mismatch alone on TIC has also been noted in other reports^22^, but the 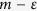 map also reveals a deviation from this trend at the upper ridge of the map.

Instead of using system properties that reflect the bulk vibrations, it was postulated that what may serve as more useful descriptors are the properties that are specifically related to the interfacial region’s vibrations. As a first hypothesis, we examined the spectrum of the vibrations near the interface (i.e., interfacial power spectra (IPS)), which have been previously observed to differ from the bulk in some structures^35^,^36^. We determined the power spectrum of the interfacial atoms at each side of the interface by calculating the averaged power spectrum of velocities^37^,^38^. The atomic velocities 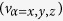 are extracted every 10 time steps (e.g., 1.5 fs), and the power spectrum is calculated using^37^,^38^,


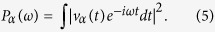


**Figure 6:**
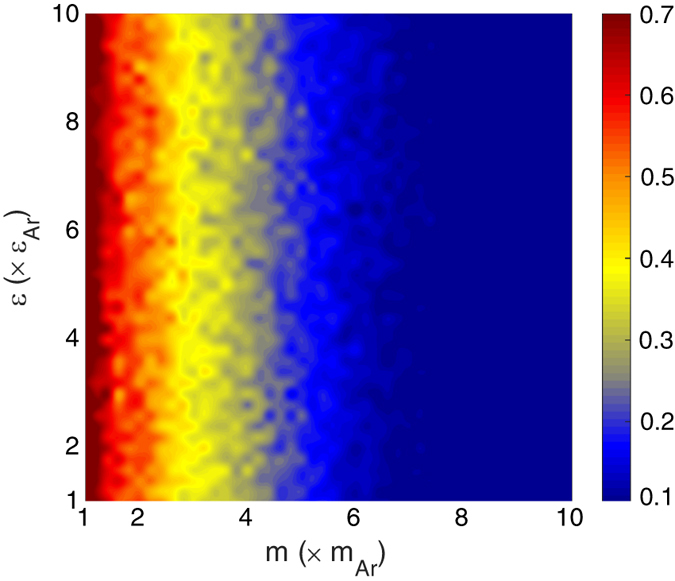
Degree of overlap between the DoS of the two sides of the interface based on the vibrations of atoms in the interface region at T = 40 K. The calculated degrees of overlap have negligible temperature dependence and show similar trends for T = 5 K, too.

Then, using Eq. 4, the degree of overlap between the interfacial DoS at the two sides of the interface is calculated for each structure and the results are shown in Fig 6. It can be seen that if we use the frequency content of the interfacial atoms, the frequency overlap (Fig. 6) differs significantly from the bulk vibrational information (Fig. 2a) and the trends are much closer to the TIC values on the 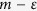 map (Fig. 5a). The fact that IPS presents a better description for heat transfer at the interface of two solids has also been reported by Chen *et al.* for the heat transport across SiGe interface, where more complex many-body potential is used in the MD simulation^36^. However, a recent study by Alexeev *et al.*^39^ showed that for the solid-liquid interface, the liquid layering effect near the interface, rather than the frequency overlap, plays an important role in determining the interfacial heat transport.

Figures 5a and 6 show that the mass ratio plays the dominant role in determining the TIC and degree of interfacial region DoS overlap on the 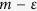 map. It was then realized that this is because even though the mass and stiffness affect the characteristic frequency in the same way, they do not affect the interfacial interactions in the same way. Most notably, as one moves from one side of the interface to the other, there is a continuous transition in the stiffness, but the transition in mass is discontinuous. Consider for example, when the stiffness on the other side of the interface is 2X larger, the atoms on the softer side, due to the use of mixing rules, experience stiffer long ranged interactions with atoms from the stiffer side that become increasingly strong as one moves toward the interface. At the interface itself, the last plane of softer atoms experiences weaker bond strength on one side, and a much stiffer bond strength on the other. Thus, as one increases the stiffness of the stiffer side of the interface, the increased stiffness also affects the atoms in the softer material and consequently makes them vibrate at higher frequencies. The net effect is that it keeps the frequency content of the atoms in the interfacial region matched for any choice of stiffness. This then leads to a fixed amount of interfacial DoS overlap, regardless of the difference in stiffness. The mass difference, on the other hand, is fundamentally different, because it does not penetrate into the other material the way the strength of interaction does. Since changing the mass difference is independent, one can decrease the frequencies of vibration for one side of the interface without changing the frequencies on the other side, thereby explaining why Figs. 5a and 6 seem to only show trends with respect to mass, but nearly constant TIC vs. stiffness ratio. Prior to this investigation, this behavior would have been non-intuitive and thus the results are highly corrective for our intuition.

The remaining discrepancy between Figs 5a and 6 is the higher TIC observed for systems with a high stiffness mismatch, for all mass ratios (e.g., the top horizontal region). It was postulated that this region of increased TIC could be a result of anharmonic effects. Testing whether this is true is straightforward, as one can suppress anharmonic effects, by probing the behavior at lower temperatures. Interestingly, when the EMD calculations are repeated at lower temperatures (5 K) the aforementioned region of higher TIC disappears and the trends are in better agreement with the trends predicted by the interfacial power spectra (IPS) overlap (see Figs 5b and 6). Thus, it appears evident that the upper ridge of higher TIC on the 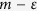 map is the result of anharmonicity (Fig. 5a). This is presumably enabled by the large asymmetry in bond strength experienced by atoms in the interfacial region. When there is a large asymmetry in stiffness, the atoms near the interface in the softer material are likely to sample the more anharmonic portions of their potential energy landscape. This is because their net forces are primarily dictated by the much higher forces from the stiffer side.

## Interfacial Power Spectrum Approximation (IPSA)

Given that the interfacial power spectrum (IPS) appears to be a more appropriate descriptor for the TIC, it is straightforward to then utilize the IPS within the DMM approach to then make improved TIC predictions. Our modification which is simply termed the interfacial power spectrum approximation (IPSA) can be employed in the traditional DMM approach^9^,^10^ as follows,


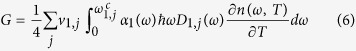


where index 1 represents the softer material, index 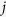 refers to the phonon polarization, 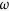 is the phonon frequency, 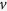 is the phonon velocity, 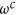 is the cutoff frequency, 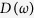 is the density of states, 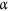 is the phonon transmission probability and 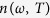 is the phonon distribution function for a mode with frequency 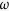 at temperature 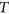. Here, specifically for comparison with the classical MD simulations, we have taken 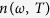 to be the classical distribution, but it should be noted that when comparing to experiments, one should use the Bose-Einstein distribution. The definition of transmission probability for a phonon with frequency 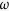 is then based on the vibrational characteristics at the interface region as opposed to the bulk,


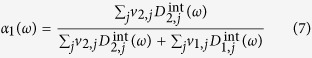


where, in addition to the parameters defined for Eq. 6 (i.e., 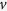 still referring to the bulk phonon velocities), index 2 represents the stiffer material, and 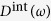 refers to the interfacial power spectra. More details on the implementation of IPSA method are provided in supplementary note 1. Information about the bulk DoS can be acquired from experiments^40^ or first-principles calculations^41^, but it is not clear how one could use these techniques to calculate the power spectra at the interface for sufficiently large systems. Thus, for the IPSA, LD calculations or MD simulations of the interface structure based on an accurate inter-atomic potential serve as the most tractable tool for determining the interfacial power spectra for a desired structure.

**Figure 7:**
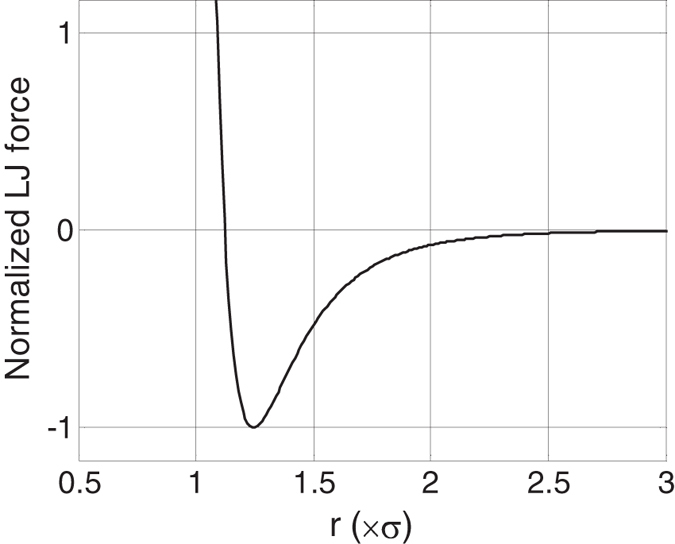
LJ force as a function of interatomic separation distance (*r*). The calculated forces are normalized based on the maximum attractive force. First, second, third, and fourth nearest neighbors in an FCC lattice are approximately located at 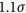, 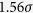, 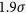, and 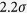, respectively^44^. The proportionality of forces at these separation distances determine the factors used to weight the power spectra of atoms at the interface in the calculation of the total averaged interfacial power spectra. For the LJ interatomic potential and FCC lattice, the weighting factors for the first, second, third, and fourth nearest neighbors were approximately 0.64, 0.31, 0.04, and 0.01, respectively.

An interesting point, associated with defining the IPS, is determining the effective region that should be termed the interfacial region. A simple approach to managing this issue is to define the vibrational power spectrum calculated for each atom with a weighting factor that is proportional to the magnitude of the force it experiences from the atoms on the other side of the interface. Figure 7 illustrates this idea by showing the forces experienced by an atom according to the LJ potential as a function of the distance between the two atoms. Although, in this study the cutoff radius has been taken as 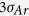, it can be seen that the first and second nearest neighbors in the FCC lattice experience much stronger interactions than subsequent neighbor shells and as a result they constitute more than 95% to the calculated interfacial power spectra. It should further be emphasized that an appropriate weighting scheme is crucial, because the vibrational spectrum quickly returns to the bulk spectrum just 2–3 layers away from the interface.

**Figure 8:**
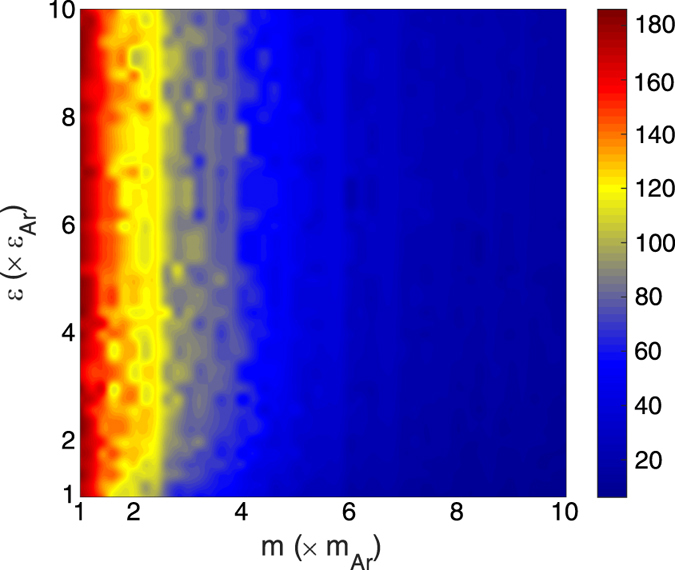
Values of TIC calculated using DMM and the interfacial DoS overlap to calculate the transmission probabilities of phonons at the interface.

As an initial test of this approach, we calculated the TIC values on the 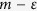 map using Eqs 6 and 7 (see Fig. 8). Compared to the traditional DMM that is based on the bulk vibrational characteristics (see Fig. 3), incorporating the IPS to calculate the transmission probabilities leads to much better qualitative agreement between the DMM calculations and the MD (see Fig. 5). However, since the rationale for why one would use frequency overlap at all, is still based on the physics of elastic interactions, even the IPSA is unable to account for the anharmonic effects observed on the upper ridge of the 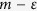 map (see Fig. 5a). Thus, although IPS is an improved descriptor as compared to bulk DoS, even for such a simple LJ system, the physics of TIC still involves anharmonicity, which is currently only well described by more involved methodologies such as the Interface Conductance Modal Analysis (ICMA) technique^20^ or recently developed spectral decomposition methods^19^,^42^,^43^.

## Summary

In this study, using equilibrium molecular dynamics (EMD), we calculated the thermal interface conductance (TIC) for over 2,000 interfaces formed at the junction of two Lennard-Jones solids. The interfaces were changed by systematically modifying the mass and stiffness on only one side of the interface, whereby the parameters on other side were held constant and corresponded to solid argon. The TIC values were also calculated using the DMM and AMM approaches, however, both of these techniques predicted drastically different trends for the variations in TIC than what was obtained by EMD simulations. Both of these approaches are based on the bulk vibrational information, and the discrepancies in the results clearly show that using the bulk vibrational information may be inaccurate for predicting interfacial transport properties. Following the postulate that interfacial vibrational properties play a key role in the interfacial heat transfer, we then showed that incorporating the vibrational power spectra of atoms at the interface into the calculation of the transmission coefficient in the DMM (i.e., interfacial power spectra approximation (IPSA) approach) significantly improved the predicted TIC values. The remaining differences between the EMD and IPSA predictions are attributed to anharmonic interactions at the interface. Incorporating such effects demands more involved techniques, such as MD based approaches^20^, that allow the inclusion of inelastic coupling between different modes of vibration.

## Additional Information

**How to cite this article**: Gordiz, K. and Henry, A. Examining the Effects of Stiffness and Mass Difference on the Thermal Interface Conductance Between Lennard-Jones Solids. *Sci. Rep.* **5**, 18361; doi: 10.1038/srep18361 (2015).

## Supplementary Material

Supplementary Information
