# Supplementary material for: Examining the Effects of Stiffness and Mass Difference on the Thermal Interface Conductance Between Lennard-Jones Solids
Source: Sci Rep. 2015 Dec 17;5:18361. doi: 10.1038/srep18361 (PMC4683623; doi:10.1038/srep18361)
Supplement: Supplementary Information [file srep18361-s1.doc]

Title: Examining the Effects of Stiffness and Mass Difference on the Thermal Interface Conductance Between Lennard-Jones Solids

Authors: Kiarash Gordiz^1^, Asegun Henry^1,2,*^

Affiliations:

^1^George W. Woodruff School of Mechanical Engineering, Georgia Institute of Technology, Atlanta GA, 30332

^2^School of Materials Science and Engineering, Georgia Institute of Technology, Atlanta GA, 30332

*Correspondence to: ase@gatech.edu

Supplementary Notes:

**Supplementary Note 1. Implementation of interfacial power spectrum approximation (IPSA)**

Implemention of the interfacial power spectrum approximation (IPSA) is essentially the same as the widely used diffuse mismatch model (DMM) ^1,2^. Therefore, we first review how the DMM was utilized to calculate the thermal interface conductance (TIC) values on the $m-\varepsilon$ map (Fig.3 in the main manuscript). As mentioned in the main manuscript, this involves several common assumptions, namely: (1) isotropic properties for both sides of the interface, (2) Debye model for the phonon dispersion and the density of states (DoS), and (3) elastic interactions between phonons across the interface. Isotropic approximation allows the following definition to be used for calculating the TIC ^2,3^,^4^

where index 1 represents the softer material, index $j$ refers to the phonon polarization, $\omega$ is the phonon frequency, $v$ is the phonon velocity, $\omega^{c}$ is the cutoff frequency, $D^{bulk}\left(\omega\right)$ is the DoS at the bulk of the material, $\alpha$ is the phonon transmission probability and $n\left(\omega,\mathrm{T}\right)$ is the phonon distribution function for a mode with frequency $\omega$ at temperature $T$. For comparison with the classical MD simulations presented in this study, we have taken $n\left(\omega,\mathrm{T}\right)$ to be the classical distribution. For all the investigated interfaces on the $m-\varepsilon$ map, the softer side is the *constant* side (i.e., the real argon side in Fig. 1 in the main manuscript). In addition, following the isotropic assumption, we simply evaluate the vibrational properties for the phonons propagating in the [100] crysalline direction, since all the simulated interfaces are chosen to be perpindicular to this direction. The phonon transmission $\alpha_{1}$ in Eq. 1 can then be calculated based on the elastic interaction approximation by,

$$\alpha_{1}\left(\omega\right)=\frac{\sum_{j} v_{2,j}D_{2,j}^{bulk}\left(\omega\right)}{\sum_{j} v_{2,j}D_{2,j}^{bulk}\left(\omega\right)+\sum_{j} v_{1,j}D_{1,j}^{bulk}\left(\omega\right)}$$

through which only the same frequency phonons are allowed to interact and exchange energy. The DOS in Eq. (2) are calculated based on the bulk vibrational information. Up to this step, this DMM formulation can be applied to any description of the phonon vibrations in the system. For example, one could use the exact dispersion curves for the solids at the sides of the interface ^3^, or one could use the more common approach of describing the dispersion and DoS with the Debye approximation ^5^.

According to the Debye approximation, the phonon velocity $v_{j}$ is taken as constant and equal to the sound velocity for each polarization type $j$ ^2,5,6^. Hence, the dispersion curve for each phonon branch/polarization $j$ would be a linear relation in the form of $\omega=v_{s,j}k$, where $\omega$ is the phonon frequency, $k$ is the phonon wave vector, and $v_{s,j}$ is the maximum group velocity (i.e., sound velocity) for polarization $j$ ^5^. In this study, we numerically evaluated slope of each polarization’s dispersion. Furthermore, the DOS at the frequency of vibration $\omega$ and polarization $j$ can be calculated by ^5^,

$D_{j}\left(\omega\right)=\omega^{2}/\left(2\pi^{2}v_{s,j}\right)$.

Lastly, the cutoff frequency $\omega_{1,j}^{c}$ for each branch can be calculated from ^5^,

$$\omega_{1,j}^{c}=v_{s,j}\left(6\pi^{2}N_{1}\right)^{1/3}$$

where $N_{1}$ is the total number of acoustic phonon modes in side 1 ^5^. For FCC lattice structures as in this study, $N_{1}$ is calculated by $N_{1}=\rho/N_{A}M$ , where $\rho$ is the mass density, $N_{A}$ is Avogadro’s number, and $M$ is the atomic weight ^7^.

The only step that should be modified for the IPSA implementation is the definition of transmission coefficient in Eq. (2), which now, instead of being based on the bulk DOS $\left(D_{j}^{bulk}\right)$, uses the interfacial DOS $\left(D_{j}^{\mathrm{int}}\right)$,

$$\alpha_{1}\left(\omega\right)=\frac{\sum_{j} v_{2,j}D_{2,j}^{\mathrm{int}}\left(\omega\right)}{\sum_{j} v_{2,j}D_{2,j}^{\mathrm{int}}\left(\omega\right)+\sum_{j} v_{1,j}D_{1,j}^{\mathrm{int}}\left(\omega\right)}$$

Therefore, all the group velocities and cutoff frequencies in the traditional DMM would remain unchanged in IPSA.

**References**

1 Swartz, E. & Pohl, R. Thermal resistance at interfaces. *Appl. Phys. Lett.* **51**, 2200-2202 (1987).

2 Swartz, E. T. & Pohl, R. O. Thermal boundary resistance. *Rev. Mod. Phys.* **61**, 605 (1989).

3 Reddy, P., Castelino, K. & Majumdar, A. Diffuse mismatch model of thermal boundary conductance using exact phonon dispersion. *Appl. Phys. Lett.* **87**, 211908 (2005).

4 Hopkins, P. E. Multiple phonon processes contributing to inelastic scattering during thermal boundary conductance at solid interfaces. *J. Appl. Phys.* **106**, 013528 (2009).

5 Kittel, C. & McEuen, P. *Introduction to solid state physics*. Vol. 8 (Wiley New York, 1986).

6 Costescu, R. M., Wall, M. A. & Cahill, D. G. Thermal conductance of epitaxial interfaces. *Phys. Rev. B* **67**, 054302 (2003).

7 Hopkins, P. E. & Norris, P. M. Effects of joint vibrational states on thermal boundary conductance. *Nanosc. Microsc. Therm.* **11**, 247-257 (2007).
